# Supplementary material for: KL-VS heterozygosity is associated with lower amyloid-dependent tau accumulation and memory impairment in Alzheimer’s disease
Source: Nat Commun. 2021 Jun 22;12:3825. doi: 10.1038/s41467-021-23755-z (PMC8219708; doi:10.1038/s41467-021-23755-z)
Supplement: Supplementary file 1 — Supplementary Information [file 41467_2021_23755_MOESM1_ESM.pdf]

KL-VS heterozygosity is associated with lower amyloid-dependent tau accumulation and memory impairment in Alzheimer's disease

Neitzel et al., Supplementary Information:

Supplementary Table 1 Main effect of global amyloid-PET levels on tau-PET levels

Cross-sectional analysis		
	**KL-VS^{het} carriers, N144**	**KL-VS^{het} non-carriers, N407**
Tau-PET SUVR, inferior temporal ROI	beta = 0.252, p = 0.002	beta = 0.441, p = 2e-16
Tau-PET SUVR, global ROI	beta = 0.279, p = 0.002	beta = 0.433, p = 2e-16
Longitudinal analysis		
	**KL-VS^{het} carriers, N52**	**KL-VS^{het} non-carriers, N148**
Tau-PET annual change rate, inferior temporal ROI	beta = 0.183, p = 0.279	beta = 0.391, p = 2.06e-06
Tau-PET annual change rate, global ROI	beta = 0.142, p = 0.401	beta = 0.275, p = 0.001

Supplementary Table 2 KL-VS^{het} × amyloid-PET interaction on regional tau-PET levels

Desikan-Kiliany region (left hemisphere)	Estimate	SE	T-value	P-value	P-value$_{[FDR-adj]}$
middletemporal	-0,0024	0,00052	-4,6615	4,88E-06	0,00016
inferiortemporal	-0,0023	0,00057	-4,1290	4,82E-05	0,00055
inferiorparietal	-0,0018	0,00053	-3,3714	0,00085	0,00484
temporalpole	-0,0017	0,00037	-4,5183	9,23E-06	0,00016
bankssts	-0,0016	0,00060	-2,6568	0,00834	0,01790
fusiform	-0,0016	0,00051	-3,0636	0,00240	0,00907
superiortemporal	-0,0013	0,00036	-3,6237	0,00035	0,00293
entorhinal	-0,0013	0,00045	-2,7880	0,00567	0,01554
parahippocampal	-0,0012	0,00037	-3,2819	0,00116	0,00565
rostralmiddlefrontal	-0,0012	0,00034	-3,4223	0,00071	0,00484
frontalpole	-0,0011	0,00035	-3,1073	0,00208	0,00886
caudalmiddlefrontal	-0,0011	0,00039	-2,7723	0,00594	0,01554
precuneus	-0,0010	0,00038	-2,7141	0,00706	0,01715
posteriorcingulate	-0,0010	0,00037	-2,6325	0,00895	0,01790
medialorbitofrontal	-0,0010	0,00032	-2,9762	0,00318	0,00981
parstriangularis	-0,0009	0,00031	-2,9932	0,00301	0,00981
lateralorbitofrontal	-0,0009	0,00036	-2,6062	0,00965	0,01822
superiorfrontal	-0,0008	0,00029	-2,6497	0,00852	0,01790

Supplementary Table 3

	ADNI tau-PET sample		ADNI amyloid-PET sample		p-value
	KL-VS^{het} carriers	KL-VS^{het} non-carriers	KL-VS^{het} carriers	KL-VS^{het} non-carriers	
N	144	407	283	784	
Age	71.29(6.61)	71.39(6.72)	72.22(6.78)	72.10(7.38)	0.221
Sex, F:M	76:68	206:201	128:123	380:404	0.745
Diagnosis, CN:MCI:ADD	102:34:8	245:122:40	133:120:30	331:343:110	<0.001[a,b,c,d]
Education, y	16.20(2.50)	16.65(2.51)	16.17(2.55)	16.40(2.57)	0.070
MMSE	28.17(2.43)	28.11(2.82)	27.97(2.39)	27.81(2.56)	0.168
ApoE ε4 status, neg:pos	90:54	255:152	163:120	454:330	0.326
Global amyloid-PET, CL	28.85(37.94)	32.28(40.30)	36.99(42.53)	38.96(43.63)	0.010[c]
Amyloid-PET status, neg:pos	89:55	232:175	146:137	400:384	0.039[c]

Group comparisons were performed using ANOVA for continuous and Kruskal-Wallis test for categorical outcome variables. Italic letters reflect the following post-hoc, pair-wise comparisons (two-sided):

[a] KL-VS^{het} carriers[tau-PET sample] versus KL-VS^{het} carriers[amyloid-PET sample],

[b] KL-VS^{het} non-carriers[tau-PET sample] versus KL-VS^{het} non-carriers[amyloid-PET sample],

[c] KL-VS^{het} carriers[tau-PET sample] versus KL-VS^{het} non-carriers[amyloid-PET sample],

[d] KL-VS^{het} non-carriers[tau-PET sample] versus KL-VS^{het} carriers[amyloid-PET sample]

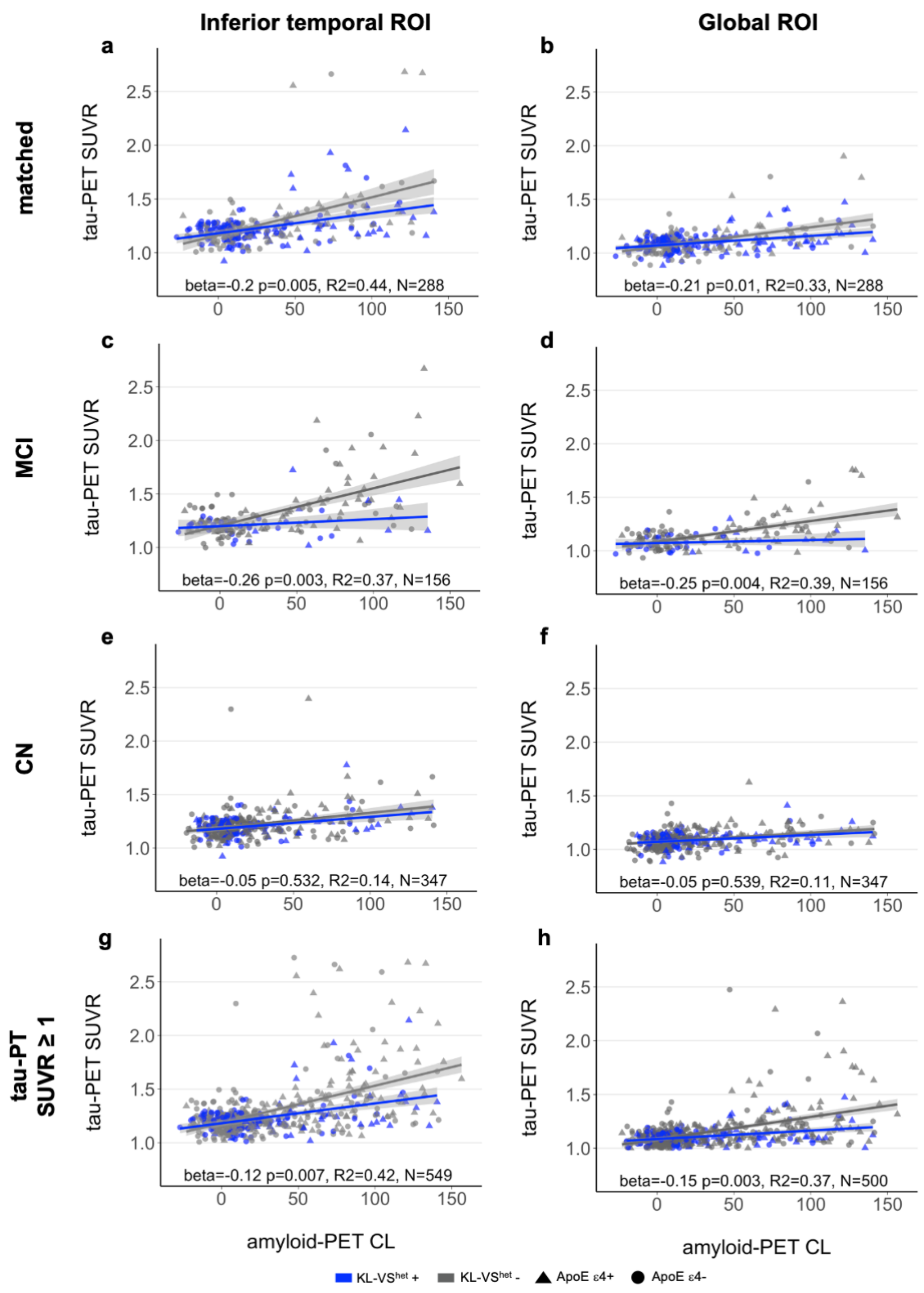

Supplementary Fig. 1 Association between KL-VS heterozygosity, amyloid- and tau-PET in different subsamples

Scatterplots display the relationship between global amyloid-PET levels and tau-PET-levels measured in inferior temporal gyri (left panel) and globally in neocortical areas (right panel) as a function of KL-VS^{het} variant in **a, b** equally sized, matched KL-VS groups (144 KL-VS^{het}

carriers versus 144 non-carriers) according to propensity scores for diagnosis and global amyloid-PET levels**, c, d** only MCI patients, **e, f** only CN participants, and **g, h** only participants with tau-PET SUVR ≥ 1. Blue and grey colour indicates individuals with heterozygous or non-heterozygous KL-VS alleles. Statistics of the KL-VS^{het} × amyloid-PET interaction effect on tau-PET uptake were derived from multiple linear regression analyses, controlled for the main effects of KL-VS^{het} status and amyloid-PET levels as well as age, sex, education, ApoE ε4 carrier status and where applicable for diagnosis. Linear model fits are indicated together with 95% confidence intervals.

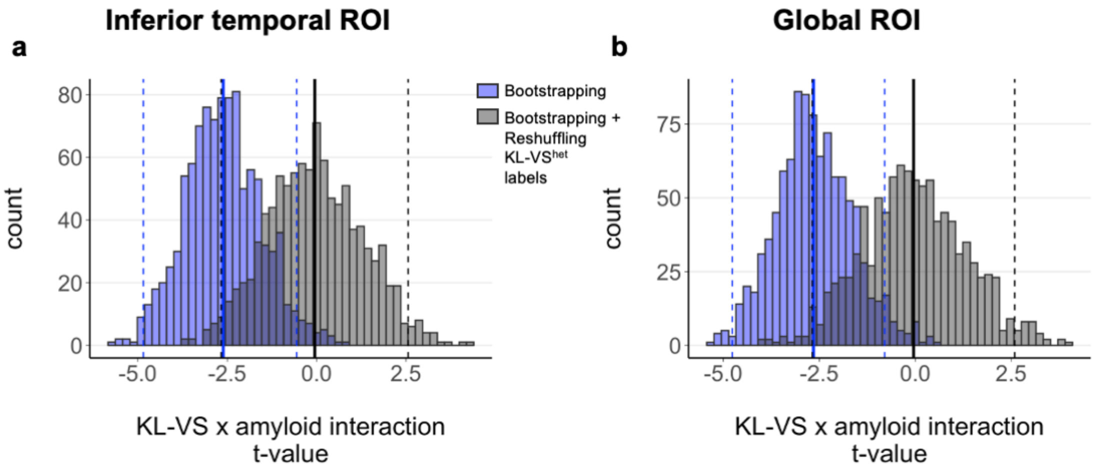

Supplementary Fig. 2 Association between KL-VS heterozygosity, amyloid- and tau-PET in 1,000 bootstrapped samples

Histograms display the distribution of the t-statistic estimating the KL-VS^{het} × amyloid interaction effect on tau-PET levels for the **a** inferior temporal ROI and **b** global ROI using 1,000 bootstrapping iterations (i.e. random sampling from the subject pool with replacement). The blue histograms show the bootstrapped results for the original data, while the grey histograms show the bootstrapped results for data in which the KL-VS^{het} labels were randomly reshuffled on each iteration. Solid lines mark the mean and dashed lines the 95% confidence intervals of the respective t-statistic distributions.

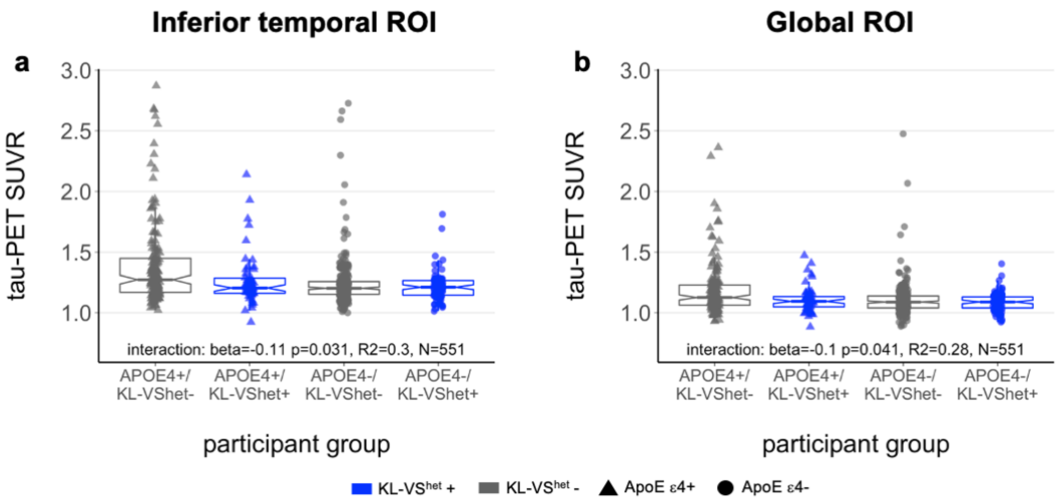

Supplementary Fig. 3 Association between KL-VS heterozygosity, ApoE ε4 carrier status and tau-PET levels

Boxplots show the relationship between tau-PET-levels measured in **a** inferior temporal gyri or **b** globally in neocortical areas as a function of KL-VS^{het} and ApoE ε4 carrier status. Blue and grey colour indicates individuals with heterozygous or non-heterozygous KL-VS alleles. Statistics of the KL-VS^{het} × ApoE ε4 interaction effect on tau-PET uptake were derived from multiple linear regression analyses, controlled for the main effects of KL-VS^{het} and ApoE ε4 status as well as age, sex, education, diagnosis and lobal amyloid-PET levels. Boxplots show the 25th percentile, median, 75th percentile (box), 95% confidence intervals of the median (notch) and 1.5x IQR (whiskers).

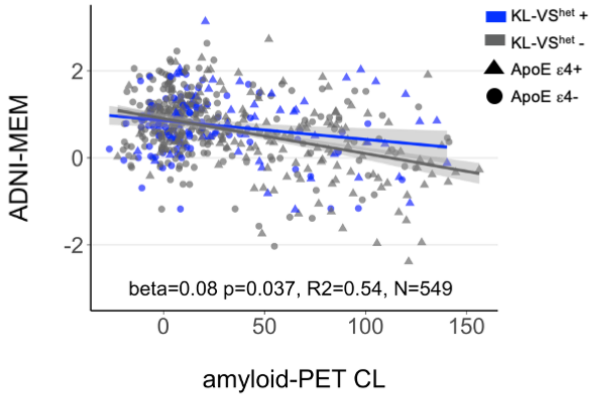

Supplementary Fig. 4 Association between KL-VS heterozygosity, amyloid-PET and memory

Scatterplots display the relationship between global amyloid-PET SUVR and memory functions dependent on KL-VS^{het} status. Blue and grey colour indicate individuals with heterozygous or non-heterozygous KL-VS alleles. Memory was measured based on an established composite score across different memory tests (ADNI-MEM). Statistical result of the KL-VS^{het} *x* amyloid-PET interaction effect on memory was derived from multiple linear regression analysis, controlled for the main effects of KL-VS^{het} and amyloid-PET levels as well as age, sex, diagnosis, education and ApoE ε4 carrier status. Two participants of the whole sample (N=551) had no ADNI-MEM scores. Linear model fits are indicated together with 95% confidence intervals.

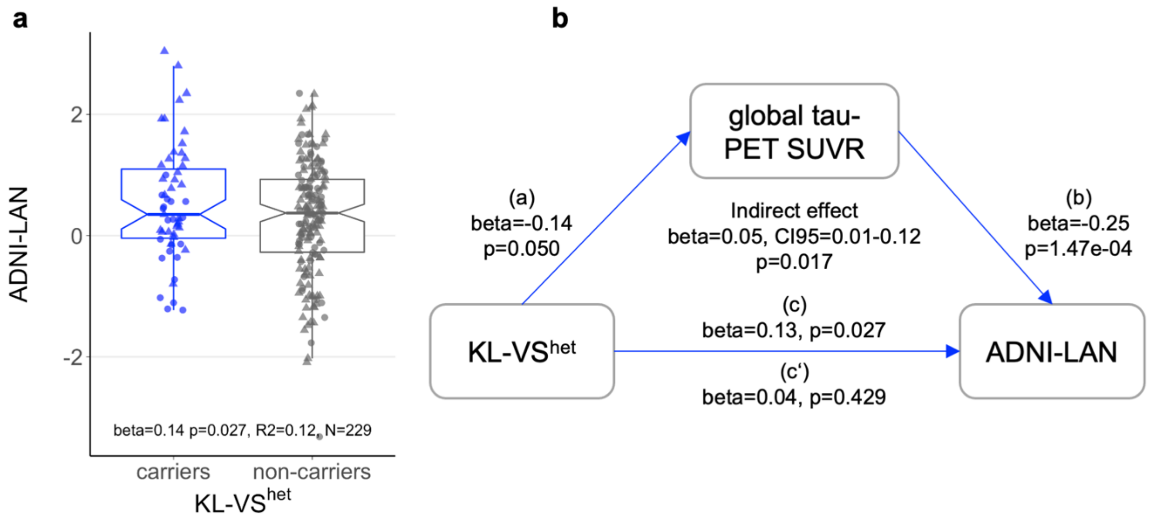

Supplementary Fig. 5 Lower tau-PET levels mediate beneficial effect of KL-VS heterozygosity on language performance in individuals with elevated amyloid-PET burden

a Boxplot shows language performance as a function of KL-VS^{het} variant in individuals with a positive amyloid-PET ($SUVR_{FBP} \geq 1.11$ or $SUVR_{FBB} \geq 1.08$). Blue and grey colour indicates individuals with heterozygous (N = 55) or non-heterozygous KL-VS alleles (N = 174). Language was measured by an established composite score, ADNI-LAN, based on test performance across multiple different language tests. Boxplots show the 25th percentile, median, 75th percentile (box), 95% confidence intervals of the median (notch) and 1.5x IQR (whiskers). **b** Path diagram of the mediation model showing that the association between KL-VS^{het} and better language performance is mediated via lower global tau-PET uptake. Path-weights are displayed as standardized beta values. All paths are controlled for age, sex, diagnosis, education, ApoE ε4 carrier status and continuous global amyloid-PET levels. Significance of the indirect effect was determined using bootstrapping with 10,000 iterations as implemented in the *mediation* package in *R*.

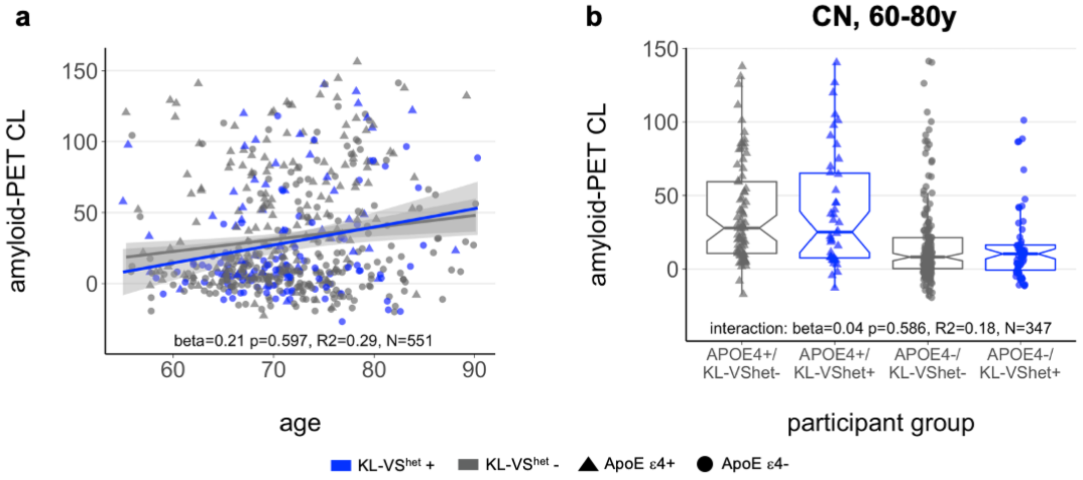

Supplementary Fig. 6 Association between KL-VS heterozygosity, age, ApoE ε4 carrier status and amyloid-PET levels

a The scatterplot displays the relationship between age and global amyloid-PET levels as a function of KL-VS^{het} status in the current ADNI sample (based on the availability of tau-PET assessment). Linear model fits are indicated together with 95% confidence intervals. **b** the boxplot displays global amyloid-PET levels as a function of KL-VS^{het} and ApoE ε4 status in a subgroup of cognitively unimpaired participants aged between 60 and 80 years, i.e. a subsample comparable to the one investigated in the original report on KL-VS^{het} effects on amyloid-PET[13]. Blue and grey colour indicates individuals with heterozygous or non-heterozygous KL-VS alleles. Statistics of the KL-VS^{het} × age or KL-VS^{het} × ApoE ε4 interaction effect on amyloid-PET uptake were derived from multiple linear regression analyses, controlled for the main effects of the interaction terms as well as sex, education, and where applicable diagnosis. Boxplots show the 25th percentile, median, 75th percentile (box), 95% confidence intervals of the median (notch) and 1.5x IQR (whiskers).

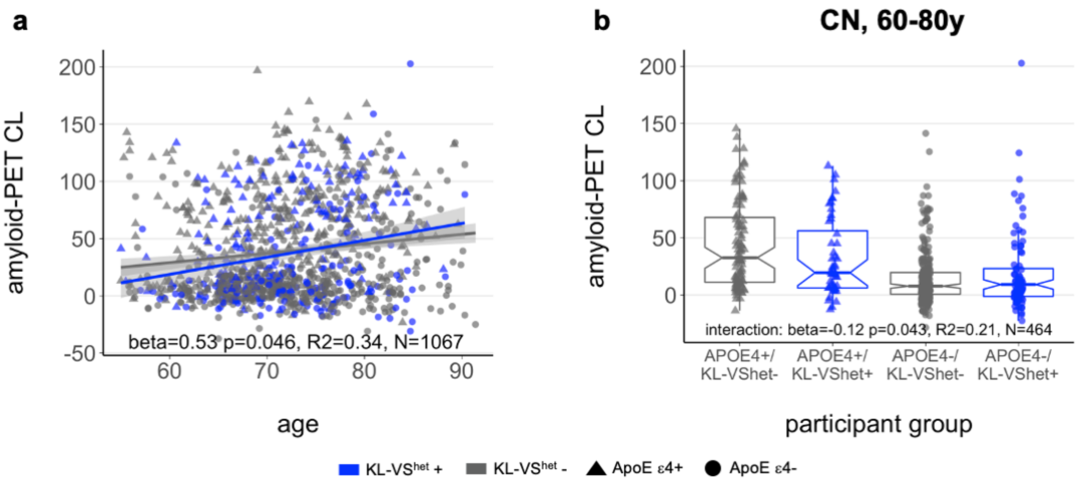

Supplementary Fig. 7 Confirmatory analyses in a larger sample: KL-VS heterozygosity effect on amyloid-PET levels

a The scatterplot displays the relationship between age and global amyloid-PET levels as a function of KL-VS^{het} status in all ADNI participants with amyloid-PET assessment (regardless of whether or not they underwent tau-PET assessment). Linear model fits are indicated together with 95% confidence intervals. **b** the boxplot displays global amyloid-PET levels as a function of KL-VS^{het} and ApoE ε4 status in a subgroup of cognitively unimpaired participants aged between 60 and 80 years, i.e. a subsample comparable to the one investigated in the original report on KL-VS^{het} effects on amyloid-PET[13]. Note that we investigated the largest possible ADNI sample for this question in order to increase power and hence the likelihood to substantiate previously reported protective effects of KL-VS^{het} against amyloid accumulation. Blue and grey colour indicates individuals with heterozygous or non-heterozygous KL-VS alleles. Statistics of the KL-VS^{het} × age or KL-VS^{het} × ApoE ε4 interaction effect on amyloid-PET uptake were derived from multiple linear regression analyses, controlled for the main effects of the interaction terms as well as sex, education, and where applicable diagnosis. Boxplots show the 25th percentile, median, 75th percentile (box), 95% confidence intervals of the median (notch) and 1.5x IQR (whiskers).
