# Supplementary material for: KL-VS heterozygosity is associated with lower amyloid-dependent tau accumulation and memory impairment in Alzheimer’s disease
Source: Nat Commun. 2021 Jun 22;12:3825. doi: 10.1038/s41467-021-23755-z (PMC8219708; doi:10.1038/s41467-021-23755-z)
Supplement: Supplementary file 3 — Reporting summary [file 41467_2021_23755_MOESM3_ESM.pdf]

nature research

Corresponding author(s): Julia Neitzel

Last updated by author(s): Apr 20, 2021

Reporting Summary

Nature Research wishes to improve the reproducibility of the work that we publish. This form provides structure for consistency and transparency in reporting. For further information on Nature Research policies, see our Editorial Policies and the Editorial Policy Checklist.

Statistics

For all statistical analyses, confirm that the following items are present in the figure legend, table legend, main text, or Methods section.

n/a	Confirmed	
☐	☒	The exact sample size (*n*) for each experimental group/condition, given as a discrete number and unit of measurement
☐	☒	A statement on whether measurements were taken from distinct samples or whether the same sample was measured repeatedly
☐	☒	The statistical test(s) used AND whether they are one- or two-sided<br>*Only common tests should be described solely by name; describe more complex techniques in the Methods section.*
☐	☒	A description of all covariates tested
☒	☐	A description of any assumptions or corrections, such as tests of normality and adjustment for multiple comparisons
☐	☒	A full description of the statistical parameters including central tendency (e.g. means) or other basic estimates (e.g. regression coefficient) AND variation (e.g. standard deviation) or associated estimates of uncertainty (e.g. confidence intervals)
☐	☒	For null hypothesis testing, the test statistic (e.g. *F*, *t*, *r*) with confidence intervals, effect sizes, degrees of freedom and *P* value noted<br>*Give P values as exact values whenever suitable.*
☒	☐	For Bayesian analysis, information on the choice of priors and Markov chain Monte Carlo settings
☒	☐	For hierarchical and complex designs, identification of the appropriate level for tests and full reporting of outcomes
☐	☒	Estimates of effect sizes (e.g. Cohen's *d*, Pearson's *r*), indicating how they were calculated

Our web collection on statistics for biologists contains articles on many of the points above.

Software and code

Policy information about availability of computer code

Data collection	Not applicable. The authors re-used collected data and therefore did not use any software for date collection.
Data analysis	All data was analyzed using R statistical software (version 3.6.1) including the following packages: mediation (version 4.5.0), matchit (3.0.2), and boot (1.3.24). R code underlying the figures is available at https://github.com/njulianeitzel/NatCommun2021_KL-VS.

For manuscripts utilizing custom algorithms or software that are central to the research but not yet described in published literature, software must be made available to editors and reviewers. We strongly encourage code deposition in a community repository (e.g. GitHub). See the Nature Research guidelines for submitting code & software for further information.

Data

Policy information about availability of data

All manuscripts must include a data availability statement. This statement should provide the following information, where applicable:

- Accession codes, unique identifiers, or web links for publicly available datasets
- A list of figures that have associated raw data
- A description of any restrictions on data availability

The data that support the findings of this study were obtained from the Alzheimer's Disease Neuroimaging Initiative (ADNI) and are available from the ADNI database (adni.loni.usc.edu) upon registration and compliance with the data use agreement. A list including the anonymized participant identifiers of the currently used sample and the source file can be downloaded from the ADNI database (tau-PET data release in May 2020; UCBERKELEYAV1451_05_12_20.csv). The Allen Brain atlas (http://human.brain-map.org) and Freesurfer-mapped transcriptomic data from the Allen brain atlas (http://figshare.com/articles/A_FreeSurfer_view_of_the_cortical_transcriptome_generated_from_the_Allen_Human_Brain_Atlas/1439749) are freely available online. Source data underlying Figs. 2 are provided with his paper.

Field-specific reporting

Please select the one below that is the best fit for your research. If you are not sure, read the appropriate sections before making your selection.

☒ Life sciences ☐ Behavioural & social sciences ☐ Ecological, evolutionary & environmental sciences

For a reference copy of the document with all sections, see nature.com/documents/nr-reporting-summary-flat.pdf

Life sciences study design

All studies must disclose on these points even when the disclosure is negative.

Sample size	We included three overlapping samples. For the cross-sectional tau-PET sample, 551 participants from ADNI phase 3 (ClinicalTrials.gov ID: NCT02854033) based on availability of KL-VS and ApoE e4 genotyping, baseline 18F-Florbetapir or 18F-Florbetaben amyloid-PET, 18F-Flortaucipir tau-PET and T1-weihgted MRI. PET and MRI had to be obtained within the same study visit. For the longitudinal tau-PET sample, we investigated 200 participants who fulfilled the previously described inclusion criteria and additionally had a followup 18F-Flortaucipir PET available. For the cross-sectional amyloid-PET sample, we included 1061 ADNI participants who fulfilled the previously describe inclusion criteria except that the availability of 18F-Flortaucipir PET was not necessary.
Data exclusions	No data was excluded
Replication	No replication in an independent dataset was performed due to lack of sufficiently large cohorts that have tau-PET, amyloid-PET imaging and KL-VS genotyping available.
Randomization	Allocation to groups was based on KL-VS heterozygosity variant, so no randomization was performed.
Blinding	Investigators were blinded to group allocation during data collection.

Reporting for specific materials, systems and methods

We require information from authors about some types of materials, experimental systems and methods used in many studies. Here, indicate whether each material, system or method listed is relevant to your study. If you are not sure if a list item applies to your research, read the appropriate section before selecting a response.

Materials & experimental systems

n/a	Involved in the study	
☒	☐	Antibodies
☒	☐	Eukaryotic cell lines
☒	☐	Palaeontology and archaeology
☒	☐	Animals and other organisms
☐	☒	Human research participants
☒	☐	Clinical data
☒	☐	Dual use research of concern

Methods

n/a	Involved in the study	
☒	☐	ChIP-seq
☒	☐	Flow cytometry
☒	☐	MRI-based neuroimaging

Human research participants

Policy information about studies involving human research participants

Population characteristics	We included three overlapping samples. For the cross-sectional tau-PET sample, 551 participants from ADNI phase 3 (ClinicalTrials.gov ID: NCT02854033) based on availability of KL-VS and ApoE e4 genotyping, baseline 18F-Florbetapir or 18F-Florbetaben amyloid-PET, 18F-Flortaucipir tau-PET and T1-weihgted MRI. PET and MRI had to be obtained within the same study visit (mean age = 71.4y (55-90), females = 51.2%, MCI = 28.3%, dementia = 8.7%, ApoE e4 carriers = 37.4%, KL-VShet carriers = 26.1%). For the longitudinal tau-PET sample, we investigated 200 participants who fulfilled the previously described inclusion criteria and additionally had a followup 18F-Flortaucipir PET available (mean age = 71.3y (55-90), females = 48.5%, MCI = 33.5%, dementia = 10%, ApoE e4 carriers = 47%, KL-VShet carriers = 26%). For the cross-sectional amyloid-PET sample, we included 1061 ADNI participants who fulfilled the previously describe inclusion criteria except that the availability of 18F-Flortaucipir PET was not necessary (mean age = 72.1y (55-91), females = 49%, MCI = 43.4%, dementia = 13.1%, ApoE e4 carriers = 42.2%, KL-VShet carriers = 26.5%).
Recruitment	All ADNI subjects were recruited within the Alzheimer's Disease Neuroimaging Initiative (ADNI, see http://adni.loni.usc.edu/) and described in Weiner et al., 2010. The authors of the study were not involved in subject recruitment. The recruitment strategy is unlikely to have an impact on this study and its finding.
Ethics oversight	Ethical approval was obtained by the ADNI investigators, all participants provided written informed consent.

Note that full information on the approval of the study protocol must also be provided in the manuscript.
